# Innovation in laboratory testing approaches to oversee Africa's local production of medical products

**DOI:** 10.1093/inthealth/ihad053

**Published:** 2023-07-29

**Authors:** Rachelle Harris, Margareth Ndmondo-Sigonda, David Mukanga, Mimi Darko, Divya Kantilal Shah

**Affiliations:** Harris Access Consulting Ltd., London E17 4PA, UK; African Union Development Agency, 230 15th Road, Midrand, South Africa; Bill & Melinda Gates Foundation, 500 5th Avenue North, Seattle, WA 98109, USA; Food and Drugs Authority, 17 Nelson Mandela Ave., Accra, Ghana; The Wellcome Trust, Gibbs Building, 215 Euston Rd., London NW1 2BE, UK

## Background

The African Union is committed to Africa becoming self-reliant in the supply of quality-assured medicines and vaccines through enhanced regulatory and manufacturing capacity. This was expressed clearly at the December 2021 meeting on Partnerships for African Vaccine Manufacturing (PAVM) in Kigali, Rwanda.^1^ The Africa Centers for Disease Control (CDC) has since developed a continental strategy and framework for action (FFA) for achieving its goal of enhancing local vaccine production to 60% of needed vaccines, from the current level of 1%, by 2040.^2^ This builds on years of interest in strengthening Africa's production capacity for medicines, manifesting in the Pharmaceutical Manufacturing Plan for Africa (PMPA), adopted in 2007. These efforts have aligned with major investments to build the necessary manufacturing capacity, including the Coalition for Epidemic Preparedness Innovations (CEPI) and the Institut Pasteur de Dakar (IPD) partnership, announced in January 2023, to provide up to US${\$}$50 million over 10 y for the manufacture of vaccines to protect against several major diseases affecting patients across Africa. Manufacturing hubs are found in Algeria, Egypt, Morocco, Rwanda, Senegal and South Africa (most active for vaccine production) and Egypt, Algeria, Morocco, Tunisia, Nigeria, Ghana, Kenya and South Africa (most active for the manufacture of finished pharmaceutical products, i.e. small molecules).^3^

Enhancing regulatory capacity and excellence on the continent to support local vaccine and medicines manufacturing is an important component of the PAVM FFA and the PMPA.

All medical products must be approved for use by the national regulatory authority (NRA) in the respective country and sufficiently quality-assured for local use and export to other markets. For vaccines, this also includes the ability to conduct regulatory lot release by the country of manufacture. To ensure that products meet internationally recognised quality standards, including demonstrated manufacturing quality, an NRA must be assessed using the World Health Organization's (WHO) Global Benchmarking Tool (GBT) and designated as having a minimum maturity level 3 (ML3) for vaccines and medicines regulation.

## Gaps and opportunities

As of May 2023, only two NRAs on the continent had been designated as being ML3 for vaccine production (Egypt and South Africa) and three for vaccine imports (Ghana, Nigeria and United Republic of Tanzania). Four are at ML3 for medicines, which does not distinguish between producing and importing (Egypt, Ghana, Nigeria and United Republic of Tanzania).^4,5^

Most African countries producing or aspiring to produce vaccines and generic medicines still lack the testing and lot release capacities needed to reach ML3, and particularly for vaccines, which require additional lot release capability.^6^ Table 1 illustrates the variable capacity leading the charge on the production agenda, as indicated by whether they have WHO pre-qualified laboratories, International Organization for Standardization (ISO) 17025 certification from the American National Standards Institute National Accreditation Board or WHO National Control Laboratory Network for Biologicals (NNB) membership.

**Table 1.** African laboratory testing capacity for medical products^10–12^

Certification	South Africa	Kenya	Ghana	Ethiopia	Mali	Nigeria	Zimbabwe	Tanzania	Uganda	Senegal
WHO pre-qualified laboratories (medicines)	√ (3)	√ (2)	√ (1)	–	–	–	√ (1)	√ (1)	√ (1)	–
WHO pre-qualified laboratories (vaccines)	√ (1)	–	–	–	–	–	–	–	–	–
ISO 17025 certification	√	√	√	√	√	√	–	–	–	–
WHO NNB membership*	√	–	–	–	–	–	–	–	–	√

Source: Based on Nwokike J, personal communication, January 2023.

*WHO NNB membership is not equivalent to accreditation.

To fulfil its potential to transition to net supply of locally produced medical products, it is imperative that Africa develop its laboratory testing resources for regulatory quality assurance on the continent. To help address capacity issues, PAVM/Africa CDC and the African Union plan to provide support to five African NRAs for regulatory oversight to strengthen their laboratory testing capacity. It will be necessary to contain the high costs involved in developing the needed capacity, particularly as costs are most substantial in the early stages of capacity building.^7,8^ Therefore, a longer-term strategic plan is needed that ensures sustainability.^9^ It is vital that countries seeking to produce medical products make better use of existing resources and engage in collaborative approaches. Such collaborative approaches have been used in Europe by the European Network of Official Medicines Control Laboratories, coordinated by the European Directorate for the Quality of Medicines & Healthcare (EDQM) to improve efficiencies and avoid duplication.^13^

### A way forward

One model that has been used in other areas of public health, including surveillance for antimicrobial resistance and wider disease areas, is the use of regional laboratory networks. Collaborative, network-based testing approaches have the potential to increase economies and efficiencies in regulatory oversight. Strategically positioning countries to access testing and lot release laboratory services from specific regional hubs is essential. Working under a common reliance framework, a single regional laboratory ‘hub’ could service several countries (‘spokes’) to support regulatory oversight in multiple countries.

The African Medicines Regulatory Harmonisation (AMRH) Initiative is working to align the various regulatory interventions on the continent with a view to minimising duplication of effort.^14^ The PAVM/AMRH proposal for the creation of an African Reliance Regulatory Laboratory Network (regional reference laboratories) for joint testing for vaccine lot release and quality assurance requirements for regulatory approval of locally produced medical products is an exciting prospect. It is founded on the concepts of regulatory reliance and recognition. For example, country A may conduct tests for quality on behalf of country B that has produced a product, and country B would rely on country A's test results to provide the necessary regulatory approval for local production in country B, and eventually for import by countries A, C and D. These concepts work towards resource optimisation and allocative efficiency, the foundation of strengthening continental regulatory systems in Africa.

## Critical success factors

This network will need to be strengthened and resourced adequately to be able to support NRAs that have not yet achieved WHO ML3 (vaccine producing) status. Plans are afoot to develop a continental reliance framework agreement to enable the legal and policy instruments to be modified in support of reliance principles and mutual recognition. Ultimately it will form an important part of the new African Medicines Agency (AMA) that is being operationalised.

To optimise resource allocation, the new network should ensure at least one accredited testing laboratory with specific assay testing competence linked to local production capacity within each regional bloc of Africa. Such a network will include laboratories that support lot release of vaccines and others supporting batch testing of small molecule pharmaceutical products, biologicals and blood and blood products, as well as post-market surveillance for small molecules. Each of these laboratories should develop a twinning partnership with producing countries to enable the rollout of the joint testing and release program. This network should also support sustained testing proficiency for relevant assays at each laboratory over time and will require standardising and updating quality control practices over time.

Applying lessons learned from the experiences of other laboratory networks, including the WHO network of laboratories for biologicals and the EDQM, will help to establish a sustainable model.^15^ Plans to strengthen capacity and develop the network would also benefit from neutral mapping of existing capacity for the various function and product categories to ensure specialised centres are created in the appropriate locations and with access to the correct resources. Accreditation of specific laboratories according to the same quality system (including ISO 17025) creates common quality standards across the network and thereby builds trust and capacity. A solid mutual recognition framework within the network and a reliance framework of accredited laboratories outside the network can increase trust and promote the use of work done elsewhere. Continued equipment maintenance, training and ongoing sharing of good practices is essential and requires sustainable financing and access to resources beyond the initial setup phase. Various laws and policies can affect laboratory practices and there may be ramifications involved in transferring services out of the country or legal barriers to relying on work done elsewhere that can prevent optimal performance of the network.^16^ Countries may also experience concern about having slower access to results if they are reliant on a laboratory elsewhere. However, with the appropriate agreements in place to sustain a framework of trust, this would enable quicker testing of small molecules and vaccines.

The African Medicines Quality Forum has been working to build and strengthen the capacity of African National Quality Control laboratories to conduct proficiency and bioanalytical testing, strengthen quality management systems and conduct post-marketing surveillance in line with the remit for regulatory monitoring as set out by the WHO GBT.^17^ Despite best efforts, until recently there has been insufficient funding for developing the required laboratory infrastructure, as well as the skills and guidance required for its effective implementation.

Additionally, as highlighted in a recent Wellcome Trust report on strengthening regulatory systems in low- and middle-income countries, the existing legal and governance frameworks, standards and procedures, human capacity and availability of sustainable financing able to support a regionally networked laboratory model are insufficient.^18^ A continentally agreed governance approach that ensures these resources are shared equitably by all countries, and ongoing capacity strengthening, will be critical success factors.

An effective, independent and transparent continental financing mechanism to support the network within the AMRH/AMA structures should be available to allow use of funds from multiple sources. This should support the network's physical infrastructure, governance and legal functions, operational delivery and capacity strengthening and twinning partnerships.^16^

Above and beyond the development of a continental reliance framework agreement, and still within the context of the AMRH/AMA structures, formal governance arrangements, such as steering boards and twinning partnerships based on reliance and mutual recognition between the countries involved, active participation and ongoing political support are essential components of success for this kind of model. The principles of this form of reliance and the results of the network should be regularly communicated. Where possible, all interventions should build on the resources already created by the AMRH and form a core part of strategic and operational planning for the AMA.

Alongside the development of suitable harmonised guidance, protocols and training, facilitation of regular communications and use of a central knowledge repository for the networks will be important to facilitate learning, alignment, information exchange and trust. Moreover, ongoing quality assurance through audits of the laboratories themselves and maintaining a supply of suitably skilled human capital and technical and financial resources will be essential. Ongoing monitoring and evaluation will be critical and should be supported by a dedicated fund.

## Conclusions

The new networks have the potential to maximise the impact of investments in public health research and development, innovation, technology transfer and manufacturing and offer value for money savings over a more piecemeal approach to capacity strengthening. By reducing barriers to regulatory reliance and ensuring targeted capacity strengthening and quality assurance, these networks will advance the mandate and implementation of the AMA agenda—and help make Africa's vision for local supply of medical products a reality.

We therefore recommend that key stakeholders extend efforts to coalesce around the proposed concept of a Reliance Regulatory Laboratory Network (regional reference laboratories) ‘hub-and-spoke’ model, as described above, that is being driven forward by the AMRH, PAVM and Africa CDC; deliver the critical success factors discussed here; and bring a range of funders and funding modalities on board, including the countries themselves.

## Data Availability

The data that support the findings of this study are derived from public domain resources and are included within the references.
